# Supplementary material for: New viruses of Cladosporium sp. expand considerably the taxonomic structure of Gammapartitivirus genus
Source: J Gen Virol. 2023 Aug 7;104(8):001879. doi: 10.1099/jgv.0.001879 (PMC10539651; doi:10.1099/jgv.0.001879)
Supplement: Supplementary material 1 [file jgv-104-1879-s001.pdf]

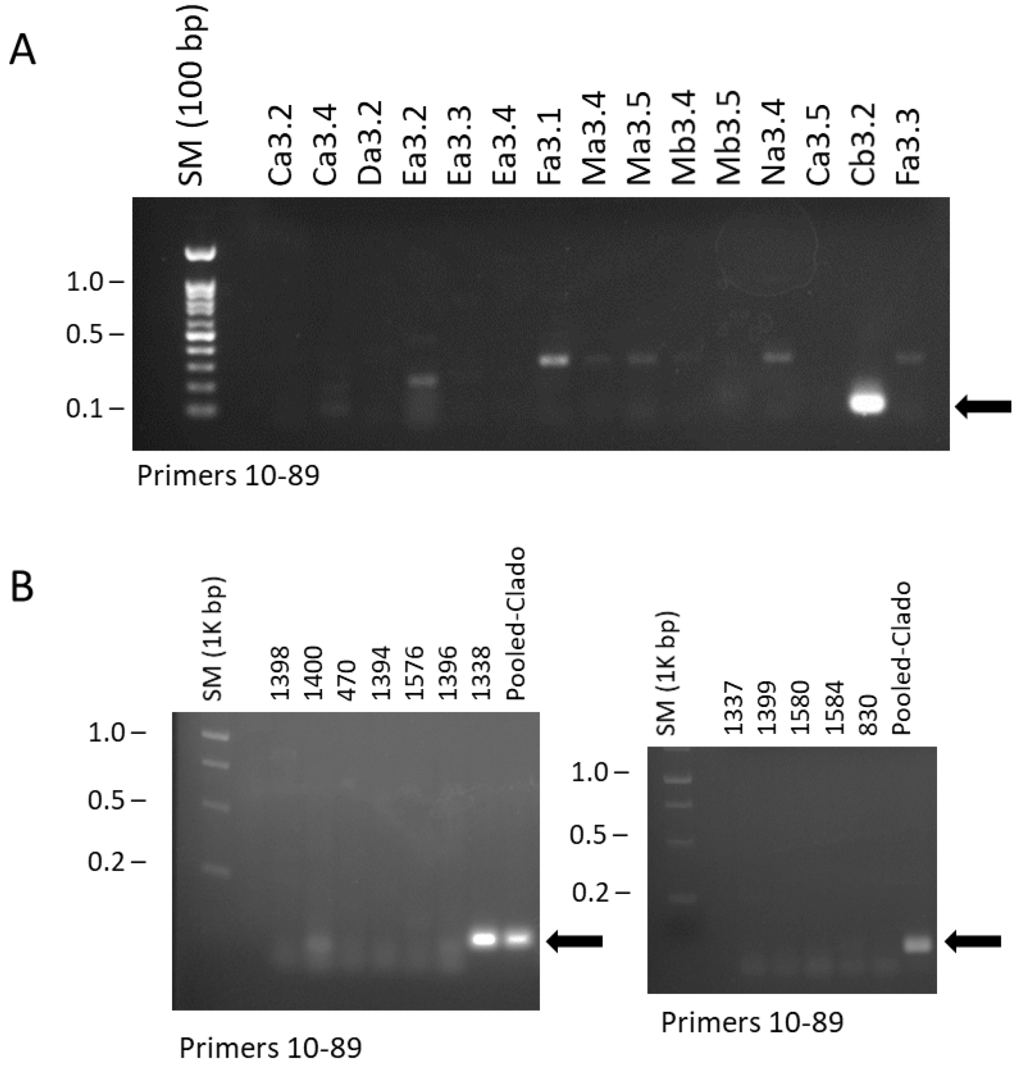

Fig. S1 : Detection of the CcPV1. RT-PCR with primer designed on the RdRp of CcPV1. The primer sequences are in the Supplementary Table S1, and expected length is highlighted with a black arrow. The size marker (SM) is a DNA ladder of 100 bp (A) and 1Kbp (B). A] Screen for the presence of CcPV1 in RNA extracts from isolates of the collection of Leytron. B] (both panels) Screen for the presence of CcPV1 in the pool of all *Cladosporium sp.* RNA extracts sent for Illumina sequencing (pooled –C. sp) of the collection of Leytron and in individual RNA extracts from Cladosporium sp. from the Agroscope collection (B).

Name	Sequence (5'-3')	Application	Sequence used for the design
2	CGCGCTTTTACCGTGTAA	RT-PCR	MN035614
10	CCTTAGTTTGCAGCACCA	RT-PCR	Contig476912-Cb3.2
89	CTCCTTTGATGTATCGTKACTT	RT-PCR	Contig476912-Cb3.2
193	TCTACTTCCTGGCAGTCC	RT-PCR	MN034127
438	GGTGGAGTGCGTCAACTA	RACE	RdRp-Cb3.2
781	ATCTTGGTGGACGCAGAT	RT-PCR + RACE	RdRp-Cb3.2
1707	CMCACACCTCAGTTTTCACT	RT-PCR	MN035614
1035	GGAAACGTGGGCATGTAC	RT-PCR	MN034127
1	ACATGGGCGAAAATTGTCC	RT-PCR	Contig1-1338
275	AGGCTCTCTCTCACCTGA	RACE	Contig1-1338
736	CGCATTGACGCTTGTTGA	RT-PCR	Contig1-1338
1349	GGTGTGGTATTTCATAAACAC	RT-PCR	Contig1-1338
2077	ACATGGGGGATTTTATAGTTTA	RT-PCR	Contig1-1338
18	GGCGAAGGCATTCACTTT	RT-PCR	Contig2-1338
121	CCTTGATCTGATCGGCGA	RACE	Contig2-1338
1087	GCCGTCTATTCTGGAGGA	RT-PCR	Contig2-1338
1245	ATGAGTCACACAGCTGGT	RT-PCR	Contig2-1338
1384	TTACCAGGGTTTTCCGCG	RACE	Contig2-1338
1637	CGGATGAAGAAGTGGCGA	RT-PCR	Contig2-1338
3033	ATGATGTAGGAGCCCAGG	RT-PCR	Contig4-1338
3133	GTGAAGTTGCATTCGGGA	RACE	Contig4-1338
3646	ACTTCTCCGGTCTCGAAA	RT-PCR	Contig4-1338
2420	CTTCTGCCTGTGCGATAA	RACE	Contig4-1338
2400	CTTATCGCACAGGCAGAA	RT-PCR	Contig4-1338
3495	GCCAATGATCTGAGCCAA	RACE	Contig4-1338
292	CTTCGGTATGCGACAGAG	RT-PCR	Contig3-1338
440	GGAGTACGAGTGTAGCCT	RACE	Contig3-1338
1081	ATCACTGGTACCCAACGA	RT-PCR	Contig3-1338
1200	GGTAACACAGCGACCATT	RT-PCR	Contig3-1338
1608	TCAAGATGCGCACTTTGT	RACE	Contig3-1338
1750	GCTTATACACGTTCGGCA	RT-PCR	Contig3-1338

Table S1: Primer used for the viral sequence detection and reconstruction.

Acronym	Accession (RdRp)	Accession (CP)	Accession (HP)	Virus name	Familly	
HPBV	BAD98236.1			Human picobirnavirus	-	
BCV1	YP_002308574.1			Beet cryptic virus 1	*Alphapartitivirus*	
CCRSaPV	AJ781401			Cherry chlorotic rusty spot associated partitivirus	*Alphapartitivirus*	
CpCV1	AM999771			Chondrostereum purpureum cryptic virus 1	*Alphapartitivirus*	
CrCV	YP_009508046.1			Carrot cryptic virus	*Alphapartitivirus*	
FvBV	AB465308			Flammulina velutipes browning virus	*Alphapartitivirus*	
HetPV1	HQ541323			Heterobasidion partitivirus 1	*Alphapartitivirus*	
HetPV3	FJ816271			Heterobasidion partitivirus 3	*Alphapartitivirus*	
RnPV2	YP_007419077.1			Rosellinia necatrix partitivirus 2	*Alphapartitivirus*	
VCV	YP_272124.1			Vicia cryptic virus	*Alphapartitivirus*	
WCCV1	AY705784			White clover cryptic virus 1	*Alphapartitivirus*	
AhV	L39125			Atkinsonella hypoxylon virus	*Betapartitivirus*	
CCCV2	JX971982			Crimson clover cryptic virus 2	*Betapartitivirus*	
CCV	ET80948.1			Cannabis cryptic virus	*Betapartitivirus*	
CpPV	YP_001911122.1			Ceratocystis polonica partitivirus	*Betapartitivirus*	
CrV1	AY603052			Ceratocystis resinifera virus 1	*Betapartitivirus*	
DCV2	JX971984			Dill cryptic virus 2	*Betapartitivirus*	
FpV1	AF047013			Fusarium poae virus 1	*Betapartitivirus*	
HetPV2	HM565953			Heterobasidion partitivirus 2	*Betapartitivirus*	
HetPV7	JN606091			Heterobasidion partitivirus 7	*Betapartitivirus*	
HetPV8	JX625227			Heterobasidion partitivirus 8	*Betapartitivirus*	
HTCV2	JX971980			Hop trefoil cryptic virus 2	*Betapartitivirus*	
PmV1	ABW82141.1			Primula malacoides virus 1	*Betapartitivirus*	
PoV1	AY533038			Pleurotus ostreatus virus 1	*Betapartitivirus*	
RCCV2	JX971978			Red clover cryptic virus 2	*Betapartitivirus*	
RnV1	YP_392480.1			Rosellinia necatrix partitivirus 1-W8	*Betapartitivirus*	
RsV	NP_620659.1			Rhizoctonia solani virus 717	*Betapartitivirus*	
WCCV2	JX971976			White clover cryptic virus 2	*Betapartitivirus*	
CSpV1	U95995			Cryptosporidium parvum virus 1	*Cryspovirus*	
BCV2	YP_009508068.1			Beet cryptic virus 2	*Deltapartitivirus*	
PCV1	YP_009466859.1			Pepper cryptic virus 1	*Deltapartitivirus*	
PCV2	YP_009351838.1			Pepper cryptic virus 2	*Deltapartitivirus*	
BbPV3	QFP40245.1			Beauveria bassiana partitivirus 3	*Epsilonpartitivirus*	
CePV1	AZT88590.1			Colletotrichum eremochloae partitivirus 1	*Epsilonpartitivirus*	
RsdRV5	AVP26802.1			Rhizoctonia solani dsRNA virus 5	*Epsilonpartitivirus*	
AflPV1	QDE53634.1		QDE53635.1	Aspergillus flavus partitivirus 1	*Gammapartitivirus*	III
AnPV1	BDF97658.1	BDF97659.1		Aspergillus niger partitivirus 1	*Gammapartitivirus*	III
AoV	ABV30675.1	YP_009665973.1		Aspergillus ochraceous virus	*Gammapartitivirus*	II
BdPV4	UVZ34178.1	UVZ34179.1		Botryosphaeria dothidea partitivirus 4	*Gammapartitivirus*	III
BdV1	AIE47694.1		AIE47695.1	Botryosphaeria dothidea virus 1	*Gammapartitivirus*	III
CaPV1	UYD21365.1		UYD21364.1	Colletotrichum associated partitivirus 1	*Gammapartitivirus*	III
CaRNAV1	AGL42312.1		AGL42313.1	Colletotrichum acutatum RNA virus 1	*Gammapartitivirus*	III
CcPV1	WEU80702.1	WEU80705.1		Cladosporium cladosporioides partitivirus 1	*Gammapartitivirus*	II
CcPV2	WEU80703.1	WEU80704.1		Cladosporium cladosporioides partitivirus 2	*Gammapartitivirus*	III
CgPV1	QED88095.1		QED88096.1	Colletotrichum gloeosporioides partitivirus 1	*Gammapartitivirus*	III
CrPV1	WEU80701.1	-		Cladosporium ramotenellum partitivirus 1	*Gammapartitivirus*	III
DdV1	AAG59816.1	NP_116742.1		Discula destructiva virus 1	*Gammapartitivirus*	II
DdV2	AAK59379.1	NP_620302.1		Discula destructiva virus 2	*Gammapartitivirus*	II

EnaPV3	QJW70322.1	QJW70321.1		Erysiphe necator associated partitivirus 3	*Gammapartitivirus*	II
EnaPV7	QJW70316.1	QJW70323.1		Erysiphe necator associated partitivirus 7	*Gammapartitivirus*	III
FcPV1	QOL02536.1		QOL02537.1	Fusarium cerealis partitivirus 1	*Gammapartitivirus*	III
FsV1	BAA09520.1			Fusarium solani virus 1	*Gammapartitivirus*	II
MoPV1	APP18151.1	APP18152.1		Magnaporthe oryzae partitivirus 1	*Gammapartitivirus*	I
MpPV1	QKO02079.1		QKO02080.1	Macrophomina phaseolina partitivirus 1	*Gammapartitivirus*	III
OPV1	AM087202	YP_009508237.1		Ophiostoma partitivirus 1	*Gammapartitivirus*	II
PnV1	YP_009551507.1	YP_009551508.1		Pythium nunn virus 1	*Gammapartitivirus*	I
PsV-F	AY738336	AAU95759.1		Penicillium stoloniferum virus F	*Gammapartitivirus*	I
PsV-S	AY156521	YP_052857.1		Penicillium stoloniferum virus S	*Gammapartitivirus*	II
PvlaPV3	QHD64801.1	QHD64799.1		Plasmopara viticola lesion associated Partitivirus 3	*Gammapartitivirus*	III/II
PvlaPV4	QHD64807.1		QHD64811.1	Plasmopara viticola lesion associated Partitivirus 4	*Gammapartitivirus*	III
ThPV2	UVB68789.1	UVB68790.1		Trichoderma harzianum partitivirus 2	*Gammapartitivirus*	III
UvMV	AGJ03719.1		AGJ03720.1	Ustilaginoidea virens mycovirus	*Gammapartitivirus*	III
UvPV	AGO04402.1	AGO4403.1	AGO04404.1	Ustilaginoidea virens partitivirus	*Gammapartitivirus*	II / III
UvPV2	YP_008327312.1		YP_008327313.1	Ustilaginoidea virens partitivirus 2	*Gammapartitivirus*	III
AaPV1	APT70073.1			Alternaria alternata partitivirus 1	*Zetapartitivirus*	-
BdPV1	AGZ84316.1			Botryosphaeria dothidea partitivirus 1	*Zetapartitivirus*	-
FePV1	QOW77954.1			Fusarium equiseti partitivirus 1	*Zetapartitivirus*	-

Table S2: Selection of viruses of the Partitiviridae family for alignment and phylogenetic tree construction.

Bright spherical particles	Dense spherical particles with a contrasted outline
29.07	40.20
28.07	38.01
32.02	35.00
31.00	33.00
33.02	36.35
34.00	38.05
33.14	39.00
29.07	38.12
31.00	36.00
34.13	39.00
29.07	40.01
34.02	39.01
30.41	40.01
27.02	34.06
30.07	34.00
33.14	33.06
34.06	33.00
36.01	37.01
33.14	36.01
28.02	31.06
30.07	35.00
30.02	34.06
34.02	37.05
33.14	34.00
	35.00

Table S3: Particle diameter (nm). Dense spherical particles with a contrasted outline and bright spherical particles were observed in strain AGS-1338 infected and measured under TEM.
